# Wire + Arc Additive Manufacturing and Heat Treatment of Super Martensitic Stainless Steel with a Refined Microstructure and Excellent Mechanical Properties

**DOI:** 10.3390/ma15072624

**Published:** 2022-04-02

**Authors:** Xiaodong Zou, Ben Niu, Linlin Pan, Jianglong Yi

**Affiliations:** Guangdong Provincial Key Laboratory of Advanced Welding Technology, China-Ukraine Institute of Welding, Guangdong Academy of Sciences, 363 Changxing Road, Tianhe District, Guangzhou 510650, China; mr.zouxd@foxmail.com (X.Z.); niub@gwi.gd.cn (B.N.)

**Keywords:** wire + arc additive manufacturing, super martensitic stainless steel, microstructure, solution + aging treatment, mechanical properties

## Abstract

Due to the advantages of relatively low cost, increased energy efficiency, increased deposition rate, and the capacity to create medium to large scale components, wire + arc additive manufacturing (WAAM) has gained growing interest. Super martensitic stainless steel (SMSS) combines outstanding strength, ductility, and corrosion resistance, making it a great option for WAAM. In the present work, an SMSS component was successfully produced by WAAM. Additionally, the influence of post-manufactured heat treatment on the microstructural characteristics and mechanical properties of SMSS components was systematically examined. A microstructural analysis of the as-printed and heat-treated samples revealed the formation of typical martensite and a small amount of retained austenite. However, the sample heat-treated by solutionizing at 1050 °C for 1 h followed by aging at 400 °C for 2 h exhibited a finer martensitic structure with an effective grain size of 5.6 μm compared to as-printed sample, leading to an increase in ultimate tensile strength from 1054 ± 6 MPa to 1141 ± 3 MPa with a concomitant increase in elongation from 7.8 ± 0.4% to 12.6 ± 0.2%. Additionally, the fracture morphology of the solution + aging sample demonstrated a more uniform distribution and greater mean size of dimples, indicating better ductility.

## 1. Introduction

Additive manufacturing (AM) technologies of metallic components have garnered considerable attention in recent years due to their several advantages over traditional manufacturing process, including cost savings, increased efficiency, and the ability to deposit complex-shaped components [1,2]. Among many AM technologies, wire + arc additive manufacturing (WAAM) is a directed energy deposition approach in which an electrical arc is dedicated to a source of heat to melt the wire feedstock and a component is deposited layer by layer [3,4,5]. In comparison to other AM technologies such as selective laser melting, WAAM provides an affordable and diverse range of wire materials, a higher energy efficiency (∼90%), the ability to build medium to large-scale components at a relatively high deposition rate, and an affordable cost [6]. A variety of metal materials have been applied in the field of WAAM technology in recent years, including aluminum alloys [7], titanium alloys [8], nickel alloys [9], and steels [10].

Due to the excellent combination of high strength, good toughness, and corrosion resistance, maraging stainless steels have demonstrated an increasing application value as engineering materials for the construction of hydraulic turbines, pump bowls, and offshore oil pipeline [11,12]. Super martensitic stainless steel (SMSS) is a novel type of maraging stainless steel with Fe-Cr-Ni-Mo system that has garnered considerable attention from researchers [13,14,15,16]. In general, the addition of 4–6 wt.% Ni could improve the strength of steel and simultaneously prevent the formation of δ-Fe by expanding the austenite phase field during the cooling [17]. 0.5–2.5 wt.% Mo addition could improve the stability of Cr in the passivation film, thereby improving the corrosion resistance of stainless steel [18]. Liu et al. [14] have produced low carbon Cr13 SMSS by vacuum induction-melting and researched the microstructures evolution and mechanical properties following various heat treatment regimes. Tensile strength and hardness were observed to diminish initially and subsequently rise as tempering temperature was raised. The influence of tempering time on the microstructure and microhardness of the 00Cr13Ni5Mo2 SMSS was illustrated by Xu et al. [19]. The results indicated that an appropriate tempering process refined the microstructure and significantly increased the hardness to almost 340 HV_0.3_.

Meanwhile, WAAM technology has been successfully applied in the field of producing maraging stainless steels components such as 17−4 precipitation-hardened (PH) stainless steels and PH 13-8Mo stainless steels due to their superior mechanical properties. Another advantage of additively manufactured maraging stainless steels is their low carbon content, which minimizes the likelihood of cracking and pore development during or after manufacturing [20]. Additionally, by carefully managing the heat treatment process, required mechanical properties of maraging stainless steels can be obtained. Caballero et al. [21] investigated the effect of various WAAM process parameters on the microstructure evolution and mechanical properties of 17-4PH stainless steel component, including shielding gas, deposition path, and post-fabrication heat treatment. Ghaffari et al. [22] examined the microstructure, microhardness, and strength of a WAAM PH 13-8Mo stainless steel component at various locations and orientations. They discovered that the volume fraction of both retained austenite and δ-ferrite gradually decreased along the bottom to the top of the component, resulting in the corresponding increment of microhardness and strength values.

However, up to now, there are very few studies concentrating on applying the WAAM process for producing SMSS. Dalaee et al. [23] focused on the feasibility of a combined direct metal deposition-plasma transfer arc welding AM technique of X3CrNiMo SMSS but provided scant information on the microstructure, mechanical properties or post-manufacturing heat treatments required to achieve satisfactory properties. During the WAAM, the deposited material experiences cyclic reheating with gradually shrinking temperature and cooling when the subsequent layers are deposited. This thermal cycle is quite different from that in the conventional wrought process and artificial heat treatment, resulting in different microstructure characteristics and mechanical properties in components produced by WAAM.

Therefore, the purpose of the present study is understanding the application of WAAM process in producing SMSS components. For that, a novel SMSS filler wire for WAAM was developed. The microstructure characteristics of WAAM SMSS have also been thoroughly explored and correlated to the mechanical properties. Additionally, the evolution of microstructure, hardness and tensile strength on account of the response to the heat treatment were studied for comparison.

## 2. Materials and Methods

### 2.1. Materials and Manufacturing Process

**Figure 1:**
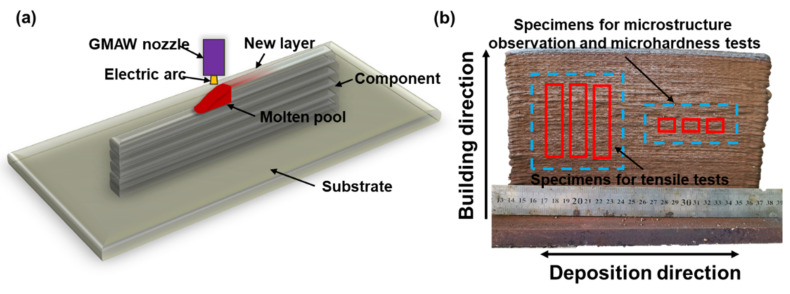
(**a**) Schematic representation of the WAAM process and (**b**) the as-printed SMSS thin-wall component.

A low carbon low alloy Q345 steel plate with a thickness of 15 mm was used as substrate to fabricate the WAAM SMSS component. The surface of this steel substrate was first mechanically ground with 600-grit emery paper and then cleaned with ethanol. A gas metal arc welding machine (Panasonic 350, Tangshan, Hebei, China) was used for the WAAM fabricated process, and the detailed welding parameters are demonstrated in Table 1. Those suitable parameters were determined based on the lowest possible spattering and on perfect bead quality with the fewest surface imperfections. Self-manufactured SMSS solid wire that was 1.2 mm in diameter was chosen as the welded material. The nominal chemical composition in weight percent was presented in Table 2. To protect the melt pool from oxidation and ambient contamination, a gaseous combination of Ar (98%) and CO_2_ (2%) with a flow rate of 20 L/min was employed as the shielding gas. The dwell duration between adjacent weld track was fixed to 3 min to avoid heat accumulation. The set-up of WAAM process is schematically depicted in Figure 1. The wire extension was set to 10 mm and maintained at this level during the whole WAAM process. As shown in Figure 1a, the multi-layer wall was fabricated by back-and-forth paths, representing the deposition direction of any two adjacent tracks were opposite. Finally, a SMSS component with dimensions of 230 mm × 160 mm × 10 mm was constructed as shown in Figure 1b.

### 2.2. Post-Manufactured Heat Treatment Process

**Figure 2:**
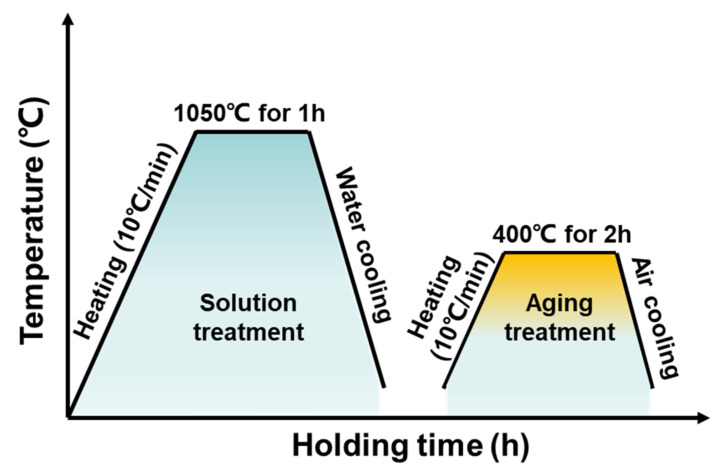
The profile of heat treatment for WAAM SMSS component.

In order to eliminate the heterogeneity of the microstructure and undesired phases, the WAAM SMSS samples were post-treated with heat treatments. Detailed heating and cooling procedures for heat treatment are listed in Figure 2. The samples were first austenitized for 1 h at 1050 °C and then water quenched to ambient temperature. Subsequently, the quenched samples were aged for 2 h at 400 °C, followed by air cooling. In addition, a direct aging treatment at 400 °C for 2 h was conducted as a baseline for comparison purposes. For convenience, we designated the as-printed sample as AP, the solution + aging-treated sample as SA, and the direct-aging-treated sample as AT.

### 2.3. Microstructural Characterization

For the microstructural studies, all specimens were cut along the building direction with dimensions of 10 mm × 10 mm × 10 mm, as shown in Figure 1b. The specimen surface from side view of component was then mechanically polished and followed by chemical etching with an etchant composed of a mixture of hydrofluoric acid, hydrochloric acid, nitric acid and water. An optical microscope (OM, ZEISS Axio Imager M2m, Jena, Freistaat Thüringen, Germany) and a scanning electron microscope (SEM, QUANTA 250, Hillsboro, OR, USA) were applied for revealing the microstructure of all samples. The phases presented in AP, AT and SA samples were analyzed by using X-ray diffraction (XRD, Rigaku SmartLab, Tokyo, Japan) with Cu Kα source at a voltage of 30 kV and a current of 135 mA. The scanning rate was set as 2°/min, ranging from 30° to 90°. Additionally, the crystallographic information and the distribution of phases were obtained by electron backscattered diffraction (EBSD) with a step size of 0.2 μm and a tilt angle of 70°. MTEX was used to analyze the volume fraction of retained austenite and crystallographic orientations through the obtained raw data.

### 2.4. Hardness and Tensile Tests

The hardness tests were conducted using a Vickers hardness tester (Wilson VH1202, Bluff Lake, IL, USA) with a diamond indenter at a load of 300 g (HV0.3) and a dwell time of 10 s, in accordance with ASTM standard ASTM-E384 (2011). Prior to microhardness test, both as-printed and heat-treated samples’ surfaces from side view were grounded by 1000-grit emery paper. An average value for at least 5 different microhardness measurements was recorded to ensure that the results are statistically available. The dog-bone tensile specimens, which were cut along the building direction of the thin wall as shown in Figure 1b, were prepared according to ISO 6892-1:2019 standard in a proportional design with 60 mm gauge length, 12 mm breadth, and 3 mm thickness to evaluate the mechanical properties. The ambient temperature tensile tests were carried out on a universal testing machine (MTS5105, Shenzhen, Guangdong, China). Fracture surfaces of samples were also examined using QUANTA 250 SEM.

## 3. Results and Discussion

### 3.1. Microstructural Characteristics

**Figure 3:**
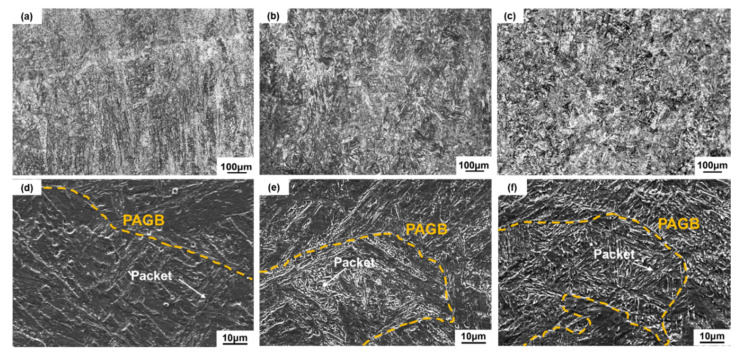
OM micrographs of WAAM samples: (**a**) AP, (**b**) AT and (**c**) SA; SEM micrographs of samples: (**d**) AP, (**e**) AT and (**f**) SA (yellow dotted lines denote prior austenite grain boundary (PAGB)).

Micrographs of WAAM and post-manufactured heat treatment SMSS samples are shown in Figure 3. From the side view, the structure of AP and AT samples is characterized by a columnar microstructure oriented along the building direction (Figure 3a,b). The solidification morphology is mainly determined by the temperature gradient (G), solidification rate (R) and cooling rate (product of G and R) [24]. In general, it is known that the columnar grain is favored when the ratio of G/R is high [25]. Therefore, in the case of WAAM, the high G/R and the absence of nucleation events upstream of the solid/liquid interface led to the occurrence of columnar grains [3]. The phenomenon is similar to other WAAM processed materials [26]. Columnar characteristics are no longer visible following the solution + age treatment (Figure 3c). To gain a better understanding of the microstructural characteristics, SEM was used. As illustrated in Figure 3d, the microstructure of the AP sample shows fully martensitic structure. A prior austenite grain is made of several martensite packets with the same habit plane in the AP specimens. After post-manufactured heat treatment, SA as well as AT samples also exhibit a characteristically martensitic structure, as shown in Figure 3e,f. The SMSS component demonstrates a very good hardenability features [27]. SA and AT samples, on the other hand, display finer martensitic laths than the AP sample.

**Figure 4:**
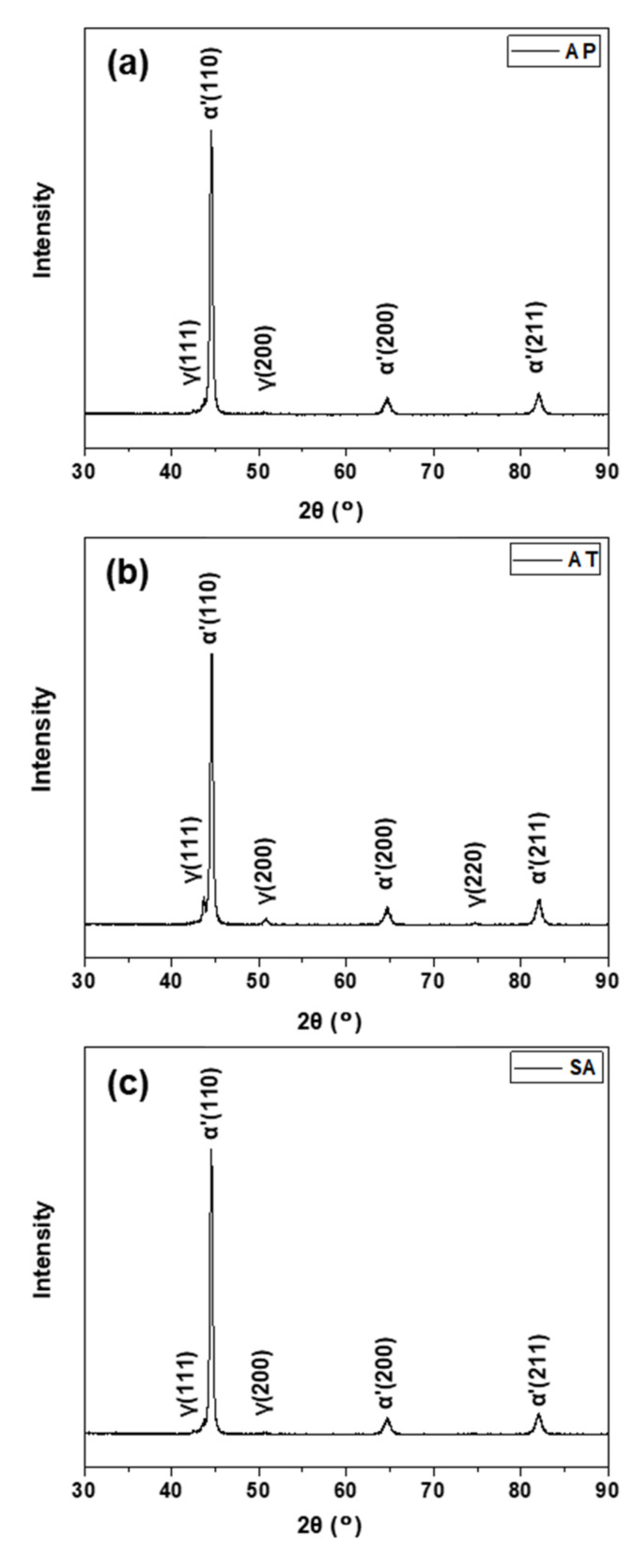
X-ray diffraction patterns of the (**a**) AP, (**b**) AT, and (**c**) SA samples.

XRD phase analysis was used to determine the presence of the second phase, i.e., retained austenite. Figure 4 shows the XRD spectrum of AP, AT and SA samples. The strong diffraction peaks of body-centered cubic (bcc) planes, such as (110), (200) and (211), corresponding to the α-Fe phase, are clearly visible in all of samples. However, the difference in the axis ratio between martensite and ferrite (10^–4^~10^–5^ nm) is extremely minor [28]. When combined with the microstructure presented in Figure 3, the diffraction peaks of α-Fe are believed to represent martensite (α’). Simultaneously, a few faint peaks of (111) and (200), representing the face-centered cubic (fcc) austenite (γ), were detected after WAAM process. The formation of austenite phase is mainly due to the consecutive heating and cooling cycles [29]. During the WAAM process, each track deposition raises the temperature of the previously deposited layer, hence increasing the diffusion of austenite stabilizing elements such as C and Ni from the supersaturated martensite to the neighboring austenite. At last, the segregation of austenite stabilizing elements results in a drop in the martensite start temperature, allowing austenite to exist at ambient temperature. After direct aging treatment, the intensities of (111) and (200) diffraction peaks significantly increase, while the (220) diffraction peak appears, indicating a greater austenite phase concentration in the AT sample. This is attributed to the development of reverted austenite during aging treatment, which generally results from complicated solute partitioning and interaction with the other microstructure and is a common occurrence during the aging treatment of maraging steel [30,31]. While the XRD result for the SA sample is identical to that for the AP sample, this indicates that the content of austenite does not change significantly during solution + aging treatment. It is well established that a high solution temperature (1050 °C) promotes the complete dissolution of the alloying elements such as C and Ni in the matrix [32]. As a result, the degree of segregation of alloying elements is reduced following solution treatment. Meanwhile, ensuing aging temperature is insufficiently high to promote the massive diffusion of austenite stabilizing elements from the martensite. Hence, only a few reverted austenites nucleate in the vicinity of the surviving austenite-stabilizing elements-enriched patches and are stable at ambient temperature [33].

**Figure 5:**
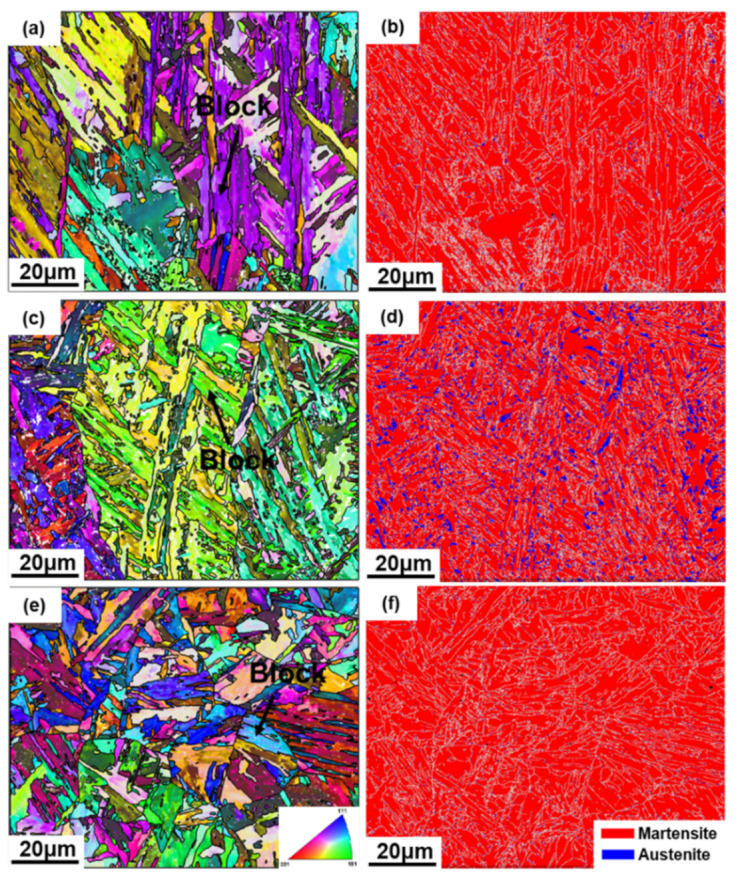
EBSD inverse pole figure maps of (**a**) AP, (**c**) AT and (**e**) SA samples, and the corresponding phase maps of (**b**) AP, (**d**) AT and (**f**) SA samples (white lines in phase maps represent grain boundaries).

To better understand the crystallographic information and the distribution of phases, the inverse pole figures (IPFs) of WAAM and post-manufactured heat treatment SMSS samples obtained by EBSD are depicted in Figure 5, which are constructed along the building direction considering the bcc iron representing the martensite phase, as well as the corresponding phase distribution maps in the AP, AT and SA samples. As illustrated in the IPFs of WAAM and post-manufactured heat treatment samples (Figure 5a,c,e), the majority of grains exhibit a mixture of crystallographic orientations roughly parallel to <001>, <101> and <111> directions. In addition, the IPFs of AP and AT samples present a coarse columnar microstructure. Moreover, all of samples demonstrate a hierarchical martensite microstructure composed of martensite blocks containing a group of laths with the same crystallographic orientation and whose boundaries frequently have high angle grain boundaries with misorientation angle greater than 15 degrees [34], embedded in different packets. Based on the EBSD study, the effective grain sizes of post-manufactured heat treatment samples (AT and SA) fall from 9.3 m to 6.8 m and 5.6 m, respectively. This demonstrates that heat-treated AT and SA samples have a finer microstructure, resulting in Hall–Petch-type strengthening [35]. During the direct aging treatment, the static recovery process may occur at 400 °C, accompanied by the interfacial segregation of alloying elements, which contributes to the formation of new interfaces within the current microstructure and promotes the refinement of grains in AT sample [36]. For SA sample, the homogenization treatment is favorable in terms of increasing the degree of columnar structure disintegration toward a finer one [26]. As a result, as illustrated in Figure 5e, a mass of new grains with approximately equiaxial forms largely replaces the previous columnar microstructure, leading to the smallest grain size.

According to the corresponding phase distribution map of AP sample (Figure 5b), roughly 2% retained austenite is visible, represented by blue color. After solution + aging treatment, the volume fraction of austenite changes to 1%, as shown in Figure 5f. Due to the diffusion of segregated C and Ni from the lath boundaries to the matrix, the retained austenite in AP samples totally transformed to martensite following the solution + aging treatment [37]. However, as illustrated in Figure 5d, the phase map of AT samples reveals a large increase in the volume fraction of austenite (10%) on account of the development of reverted austenite over martensite lath boundaries. It is worth noting that the analysis results of EBSD phase distribution maps are compatible with XRD results.

### 3.2. Hardness

**Figure 6:**
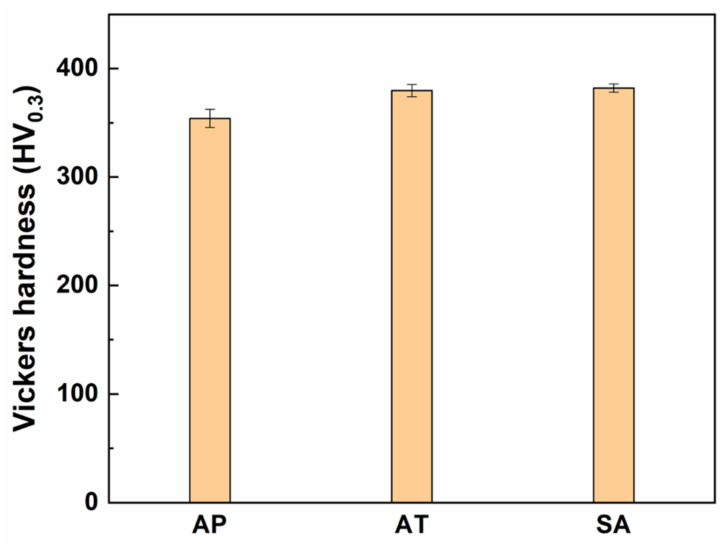
Vickers hardness measurements for AP, AT and SA samples.

Vickers microhardness measurements were initially performed to determine the heat treatment-hardening response of the current SMSS component. The variation of Vickers hardness values for WAAM and post-manufactured heat treatment samples is presented in Figure 6. The AP sample with a martensite microstructure and a limited quantity of retained austenite has the lowest hardness value of 354 ± 8 HV_0.3_. After direct aging and solutionizing + aging heat treatments, the hardness values are improved to 379 ± 6 HV_0.3_ and 382 ± 4 HV_0.3_, respectively. The increase in hardness seen in AT and SA samples can be mainly associated with the refinement of grains and secondary precipitation during aging treatment [19]. The hardness value in SA samples is slightly larger than that in AT samples due to the increased volume proportion of austenite, as illustrated in Figure 5d and f, which results in matrix softening [38].

### 3.3. Tensile Properties

**Figure 7:**
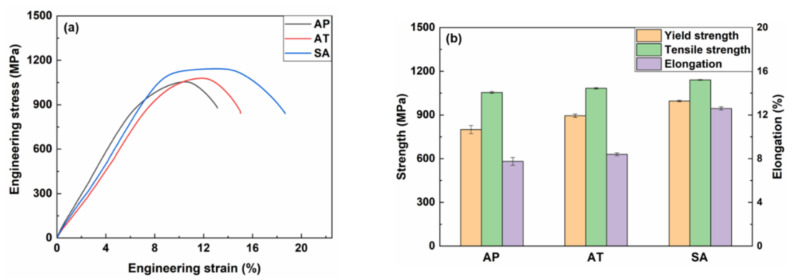
(**a**) Engineering stress-strain curves; and (**b**) the corresponding yield strength, tensile strength and elongation values of AP, AT and SA samples.

To further investigate the evolution of mechanical properties, tensile tests were conducted at ambient temperature and the acquired engineering stress-strain curves are depicted in Figure 7a, from which the yield strength, ultimate tensile strength and elongation are measured and presented in Figure 7b. The AP sample exhibits a yield strength of 800 ± 28 MPa, an ultimate tensile strength of 1054 ± 6 MPa and an elongation of 7.8 ± 0.4%. The value of strength is comparable to that reported by Ma et al. for 13Cr SMSS after solution + aging treatment [13]. During the layer-to-layer WAAM process, a portion of deposited metal near the melt pool could always be briefly reheated to aging temperature (400 °C). This may lead to a transient aging effect, resulting in the diffusion of alloying elements and the precipitation of second phase to strengthen the AP sample. However, due to the short dwell duration and absence of steady temperature, this transient aging effect is not equivalent to a proper aging treatment. After direct aging at 400 °C for 2 h, the yield strength improves to 895 ± 11 MPa, about 100 MPa more than the AP sample, and the ultimate tensile strength reaches to 1083 ± 6 MPa. Meanwhile, the ductility does not decrease, increasing to 8.4 ± 0.2%. Furthermore, the mechanical properties of SA sample demonstrate a considerable improvement in strength and ductility, with the yield strength rising to 996 ± 6 MPa, ultimate tensile strength increasing to 1141 ± 3 MPa and the elongation reaching to 12.6 ± 0.2%. Compared to the SMSS component produced by conventional approach such as hot rolling followed by heat treatment, the ultimate tensile strength in SMSS produced by WAAM is significantly improved. For instance, Ma et al. [27] reported that the maximum ultimate tensile strength in hot-rolled SMSS followed by solution treatment at 1050 °C for 0.5 h and tempering treatment at the range of 550 °C to 700 °C for 2 h could reach to about 1040 MPa, which is lower than that even in AP sample. To improve mechanical properties, varying contents of Cu were added into the SMSS by Ye et al. [39]. It was found 3 wt.% Cu-alloyed SMSS after heat treatment exhibited the highest tensile strength (near 900 MPa) and the best ductility (about 20% elongation). It is worth noting that despite the increased strength of SMSS produced by WAAM, the ductility is lower than that of conventionally fabricated SMSS. This is mainly attributed to the massive formation of retained austenite, which is particularly effective to improve the ductility and toughness, though at the expense of strength [40], at martensite lath boundaries and within laths in conventionally fabricated SMSS.

It is noteworthy that there is a noticeable variation in the performance of WAAM and post-manufactured heat treatment SMSS under hardness test and tensile loading, which can be related to their distinct microstructural characteristics. The AP sample is composed of coarse martensite as the matrix with negligible retained austenite, while direct aging and solution + aging treatment samples have a finer lath martensitic matrix with a small quantity of reverted austenite. Not only does microstructure refinement increase hardness and strength, it also plays a critical role in preventing crack propagation, hence increasing the ductility of the sample [41].

**Figure 8:**
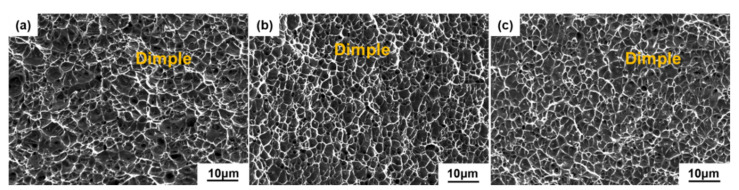
SEM morphologies of tensile fracture surfaces: (**a**) AP, (**b**) AT and (**c**) SA samples.

**Figure 9:**
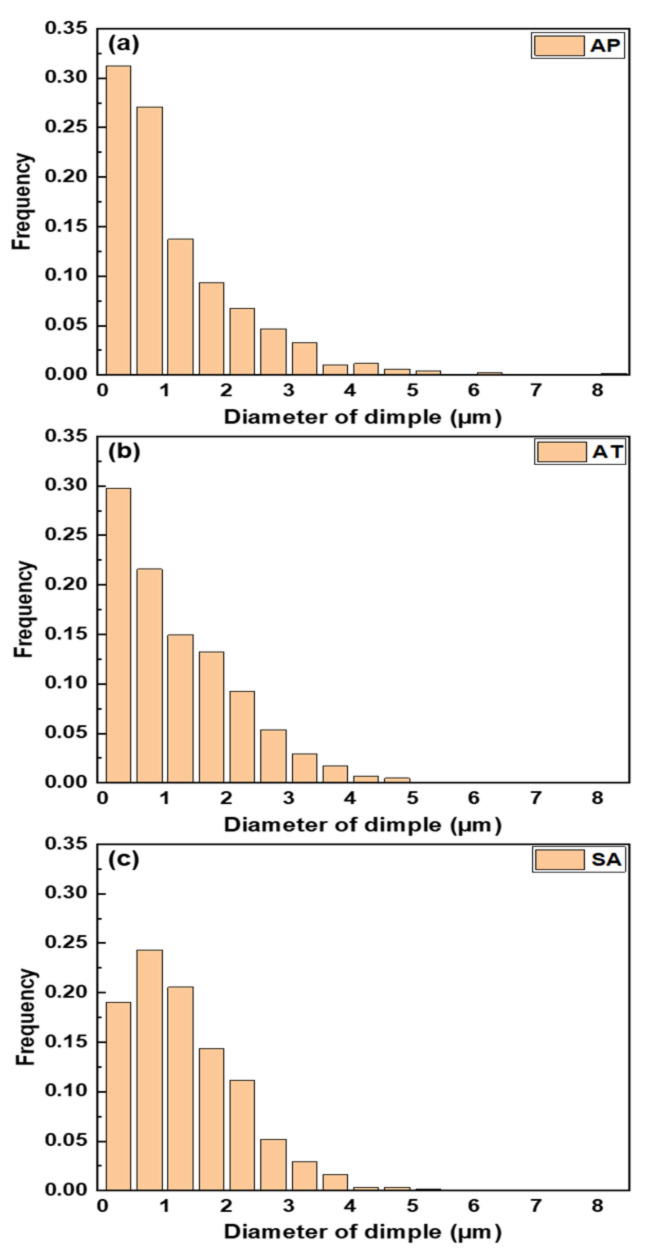
Dimple size distribution of tensile fracture at (**a**) AP, (**b**) AT and (**c**) SA samples.

Figure 8 illustrates the fracture surfaces following a tensile test in order to better understand the tensile fracture mechanism. All of samples present a high density of dimples without microcracks scattered on the fracture surfaces and have failed through voids coalescence, which are obvious features of ductile fracture. In addition, it can be observed that the dimples become more irregular in size at the AP sample compared to the AT and SA samples. The linear intercept approach was used to determine the diameters of dimples. The size distributions of dimples in all samples are shown in Figure 9, which indicate that the mean diameters of dimples in AP, AT and SA samples are 1.19 ± 1.11 μm, 1.23 ± 0.96 μm and 1.33 ± 0.83 μm, respectively. Among them, the SA sample exhibits a more homogeneous distribution and bigger mean dimple size. The primary reason may be related to the fact of fine microstructure in SA sample; consequently, the slip was prominent during deformation, leading to the formation of large dimples [42]. It is indicated by many studies that tensile ductility increases as dimple size grows size [43]. Therefore, larger dimples on the fracture surface result in the increase in elongation for SA sample. Additionally, it is widely known that the occurrence of ductile fracture contains three distinct events, namely, void nucleation, and growth and coalescence to form a crack, followed by final fracture [44]. Typically, prior austenite grain boundaries, martensite grain boundaries, and precipitates serve as nucleation sites for the voids. As a result, the finer the effective grain size in the SA sample, as illustrated in Figure 5e, the more nucleation sites for voids [45]. During the deformation, the subsequent void coalescence consumes more energy, leading to increased ductility [46].

## 4. Conclusions

In the study, the WAAM technique was successfully applied to producing full, dense SMSS components. After that, the WAAM component was heat-treated through a standard procedure consisted of solution treatment at 1050 °C for 1 h and aging treatment at 400 °C for 2 h. Moreover, a 2-h direct aging treatment at 400 °C was performed on the WAAM component. The microstructural evolution and mechanical properties of as-printed and heat-treated samples were systematically investigated, and the following findings can be drawn:

(1) The microstructure of the AP sample is formed of martensite with very limited retained austenite. After direct aging treatment, the volume fraction of austenite phase grows to approximately 10% due to the complicated solute partitioning and interaction with the other microstructure. In the SA sample, the microstructure is greatly refined as a result of a mass of freshly fine grains with roughly equiaxial forms largely replacing the prior columnar microstructure during the homogenization treatment.
(2) The solution + aging treatment has a beneficial effect on the hardness and strength of WAAM SMSS sample. The enhancement of strength in the SA sample can be attributed to the microstructure refinement. The hardness and ultimate tensile strength in the SA sample increase to 382 ± 4 HV0.3 and 1141 ± 3 MPa, respectively.
(3) All samples exhibit characteristic ductile fracture. In comparison to the AP sample, the fracture morphology in the SA sample exhibits a more homogeneous distribution and a larger mean size of dimples. In addition, the SA sample with a finer microstructure burns more energy during the deformation, resulting in better ductility.

## Figures and Tables

**Table 1:** WAAM processing parameters for the manufacturing of the SMSS wall.

Arc Current	Arc Voltage	Traveling Speed	Gas Flow Rate	Deposition Strategy
220 A	23.5 V	0.5 m/min	18 L/min	Oscillated pass

**Table 2:** Nominal chemical composition of the SMSS feedstock solid wire (wt.%).

C	Si	Mn	Cr	Ni	Mo	Cu	Fe
≤0.04	0.42	0.84	13.23	5.55	0.81	0.32	Bal.

## Data Availability

Data sharing is not applicable for this paper.
